# Hedonic hunger and food cravings: understanding their role in premenstrual syndrome among nursing students

**DOI:** 10.3389/fpubh.2025.1659974

**Published:** 2025-08-08

**Authors:** Emine Kocyigit, Mehtap Gumusay, Yagmur Demirel Ozbek

**Affiliations:** ^1^Department of Nutrition and Dietetics, Faculty of Health Sciences, Ordu University, Ordu, Türkiye; ^2^Department of Gynecologic and Obstetrics Nursing, Faculty of Health Sciences, Ordu University, Ordu, Türkiye; ^3^Department of Nutrition and Dietetics, Faculty of Health Sciences, Recep Tayyip Erdogan University, Rize, Türkiye

**Keywords:** emotional eating, food cravings, hedonic hunger, nursing, premenstrual syndrome

## Abstract

**Background:**

Premenstrual syndrome (PMS), characterized by physical, psychological, and behavioral symptoms occurring during the luteal phase of the menstrual cycle, affects more than 48% of women of reproductive age worldwide. The aim of the research is to examine the relationships between hedonic hunger, food cravings, and emotional eating in relation to PMS among Turkish female nursing students.

**Method:**

This cross-sectional and descriptive study was conducted on 207 female undergraduate nursing students. Data were obtained using survey and a face-to-face interview method. The questionnaire includes general information, anthropometric measurements, the Premenstrual Syndrome Scale (PMSS), the Power of Food Scale (PFS), the Food Craving Questionnaire-Trait (FCQ-T), and the Emotional Eater Questionnaire (EEQ). Data analysis was performed with IBM SPSS V26 software.

**Results:**

In total, 169 (81.6%) PMS (+) and 38 (18.4%) PMS (−) female students participated in the study. The mean age was 21.09 ± 2.41 years, and the mean body mass index was 23.3 ± 4.07 kg/m2. The PFS-Tr, FCQ-T and EEQ total scores was positively correlated with PMSS scores. The strongest predictor for hedonic hunger was food cravings, whereas hedonic hunger, PMS, and emotional eating were significant factors for food cravings. PMS was a problem experienced by most of the students. The results indicate that the presence of PMS is associated with increased hedonic hunger, food cravings, and emotional eating tendencies among university students.

**Conclusion:**

Raising awareness of PMS and conducting nutrition-related trainings for university students would help them get the knowledge and skills they need to manage its symptoms.

## 1 Introduction

“Hedonic hunger” describes increased appetite when metabolic needs are absent. This increased appetite is brought on by the urge to consume despite the inaccessibility of certain foods in connection with the expectation of food-related pleasure (1). Hedonic food consumption leads to excessive eating behaviors that exceed the body’s requirements, often as a means to suppress or respond to emotional states. This phenomenon is characterized by the enjoyment of palatable foods and is influenced by motivational factors or reward-punishment dynamics (2). Foods that are commonly consumed have a high energy density, along with higher amounts of salt, sugar, and fat (3). Numerous factors, including physiological differences, dietary habits, sensitivity to environmental, nutritional cues, perceived food rewards, food availability, cravings for food, emotional eating, sleep, physical activity, social media, and individual self-esteem, influence hedonic hunger (4–6).

Another factor that may be related to hedonic hunger is premenstrual syndrome (PMS). PMS is defined by a range of emotional symptoms, such as depressed mood, irritability, and anger outbursts, alongside physical symptoms, including muscle and joint pain, headaches, indigestion, and breast tenderness. Additionally, it encompasses behavioral changes like changes in eating habits, social withdrawal, and impaired concentration, primarily affecting young and middle-aged women (7, 8). Research indicates that the prevalence of PMS ranges from 24.6 to 53%. This variation predominantly results from differing diagnostic criteria, study populations, and socio-cultural factors (9–13). The worldwide incidence of PMS has been reported at 47.8% (14). The precise mechanisms underlying the pathophysiology of PMS remain unclear. Variations in sex steroid levels such as elevated estrogen levels, alterations in the estrogen-progesterone ratio, and excessive prolactin release, along with modifications in central neurotransmitters such as serotonin, gamma-aminobutyric acid, glutamate, and beta-endorphins, as well as genetic origins, biological variables, and psychological difficulties, significantly contribute to the pathogenesis of PMS (15–17). According to previous research, women with severe PMS are more prone to overeat during the luteal phase and consume high-calorie food, which may serve as a coping strategy for psychological stress (18–20). PMS influences eating behaviors, characterized by symptoms including mood fluctuations, decreased energy levels, and increased appetite (18).

Dietary patterns and nutritional status are regarded as crucial components and represent the most modifiable factor in the management of PMS (21, 22). Studies showed that increased consumption of energy-dense, fatty, and salty foods has been associated with more significant onset and exacerbation of PMS symptoms (23, 24). Carbohydrate consumption peaks during the premenstrual phase (25), alongside an increase in cravings for sweet and fatty foods (26). Furthermore, lifestyle variables and nutritional deficiencies, including sleep duration, caffeine and alcohol consumption, smoking, and elevated body mass index (BMI), are significantly correlated with PMS (10, 27). During the premenstrual phase, women are more sensitive to food stimuli, suggesting that it may be difficult to maintain regulated eating (28). Cravings for foods that release serotonin and premenstrual cravings may be exacerbated by elevated metabolism, highest progesterone and estrogen levels, and lowered serotonin levels (29). A different explanation is that women desire prohibited foods that they perceive as unhealthy and are limited to a healthy feminine diet (26). Menstrual hormonal fluctuations and physiological and neurochemical factors are associated with increased food consumption and elevated food cravings in the premenstrual phase (29, 30).

Hedonic hunger demonstrates theoretical and empirical connections with other recognized eating variables, including uncontrolled eating, which reflects opportunistic consumption when palatable food is accessible, and food craving, which signifies reward reactivity to external stimuli (5). For these reasons, determining a balanced relationship between hedonic hunger, food cravings, emotional eating, and PMS is significant, especially for university students. Extensive studies have explored the individual relationships among personality traits, PMS, nutrition intake, and eating behaviors; however, a scarcity of research has collectively examined these variables within a comprehensive model. This study aims to improve understanding of the complex interactions among variables, including menstrual cycle characteristics, anthropometric measures, hedonic hunger, food cravings, emotional eating, and PMS in university students. It aims to contribute to the literature on the diverse factors influencing eating behaviors in female university students.

## 2 Materials and methods

### 2.1 Study design and participants

The population of this cross-sectional study consisted of 362 female students studying at the Nursing Department of Ordu University in the Black Sea Region of Türkiye, between January and June 2024. A total of 207 female students who agreed to participate in the study and met the inclusion criteria were included in the study sample. A sample with a known universe was calculated for the sample, and the prevalence of PMS in university students in the study of Erbil and Yücesoy (13) was taken as a reference, as 50.3% (*p* = 0.503).

Sample calculation with a known universe:

n = [Nt^2^pq]/[d^2^(N − 1) + t^2^pq].

N: Number of individuals in the population.

n: Number of individuals to be sampled.

p: Frequency of the occurrence of the investigated event (0.503).

q: Frequency of the nonoccurrence of the investigated event (0.497).

t: Theoretical value found from the t table at a certain degree of freedom and detected an error level (1.96).

d: The desired deviation according to the incidence of the event (0.05).

It was calculated as n = [362 x (0.503) x (0.497) x (1.96^2^)] / [361 x (0.0025) + (1.96^2^) x (0.503) x (0.497)] = 187.

However, more participants were included in the study due to the possibility of leaving the study. The study was completed with 207 students.

The inclusion criteria were studying at Ordu University Faculty of Health Sciences, Department of Nursing, being female, not having a history of psychiatric illness or serious trauma in childhood, not having severe acute or chronic diseases, and not using oral contraceptives. The exclusion criteria were the inability or reluctance to complete the questionnaire, being pregnant or breastfeeding. Participants who began the research but decided not to proceed for any reason, those with communication difficulties, or people who provided insufficient information were eliminated from the study. The research was carried out following the Declaration of Helsinki. Informed consent was obtained from the participants.

### 2.2 Data collection tools

#### 2.2.1 General information and anthropometric measurements

The research data were obtained through face-to-face interviews via a questionnaire prepared by the researchers. The general information form included questions evaluating sociodemographic characteristics (age [year], grade of education, monthly income, and living situation), health problems (yes/no), menarche age (year), menstrual cycle (day), current smoking status (yes/no), and number of main meals and snacks.

The body weights were obtained in the morning with the individual fasting, barefoot, and wearing lightweight clothes using the Tanita BC 601 Inner Scan. We used a portable stadiometer to measure height to ensure that the individuals stood upright and their heads aligned with the Frankfurt plane (31). Anthropometric measurements of the students were taken during non-menstrual periods based on their self-reports. The height was measured with a precision of 0.1 cm, while the weight was measured with an accuracy of 0.5 kg. The body mass index (BMI) was calculated using the formula BMI (kg/m^2^) = weight in kg/height in meters (32). The BMI values were categorized based on the guidelines provided by the World Health Organisation (33).

#### 2.2.2 Premenstrual syndrome scale (PMSS)

The premenstrual syndrome scale (PMSS), developed by Gençdoğan in 2006 (34), was created to measure the severity of premenstrual symptoms. It is a five-point Likert scale with 44 questions and nine subdimensions. The nine subdimensions of the PMSS are Depressive mood, Anxiety, Fatigue, Irritability, Depressive thoughts, Pain, Appetite changes, Sleep changes, and Bloating. The Premenstrual Syndrome Scale facilitates a retrospective assessment of symptoms occurring “within 1 week before menstruation.” The scale is evaluated with the following points: “never” corresponds to 1 point, “rarely” to 2 points, “sometimes” to 3 points, “frequently” to 4 points, and “always” to 5 points. The PMSS score ranges from 44 to 220 points, with 110 points as the diagnosis’s cutoff. In the analysis of PMSS outcomes, PMS is considered to be present when over 50% of the subscales show high values. Therefore, those with a PMSS total score beyond 50% (> 110) were classified as PMS (+), whereas those with a total score below 50% (< 110) were classified as PMS (−). The reliability coefficient, determined by Cronbach’s alpha, was found to be 0.75 for the overall scores of the scale, while the subscales exhibited values ranging from 0.75 to 0.91.

#### 2.2.3 Power of food scale-Turkish version (PFS-Tr)

The power of food scale was created by Lowe et al. (35) to evaluate the psychological effects of residing in food-abundant surroundings, specifically the assessment of hedonic hunger state. The Turkish validity and reliability of the scale were conducted by Ülker et al. (36). Five-point Likert scales are used for the evaluation (1 strongly disagree, 2 disagree, 3 uncertain, 4 agree, and 5 strongly agree). The PFS-Tr scale consists of 13 items categorized into three subscale: food available (FA), food present (FP), and food tasted (FT). The first subscale is FA, which evaluates general perceptions regarding food (items 1, 2, 9, 10). Second, the FP subscale includes questions (items 3, 4, 5, and 6) that evaluate the attractiveness of food to which the person has direct access. The third subscale, the FT subscale, has items that evaluate the desire and enjoyment derived from food during initial tasting, before to consumption (items 7, 8, 11, 12, and 13). Total and subscale scores are calculated by summing the item scores and dividing by the total number of items. The maximum possible score on the scale is 5, while the lowest is 1. A higher score indicates an increased tendency for hedonic hunger. The Cronbach’s alpha coefficient for PFS-Tr was 0.92, while the values for “food available,” “food present,” and “food taste” were 0.85, 0.80, and 0.82, respectively (36).

#### 2.2.4 Food Craving Questionnaire-Trait (FCQ-T)

Cepeda-Benito et al. (37) developed the Food Cravings Questionnaires-Trait (FCQ-T) scale in the United Kingdom in 2000 to assess the frequency and intensity of general food craving experiences. In 2017, Müftüoğlu et al. carried out the validity and reliability assessment of the FCQ-T in Turkey. The FCQ-T has nine sub-factors and 39 items. A minimum of 39 points and a maximum of 234 points can be obtained from the FCQ-T (38). Sub-factor 1: having intentions and plans to consume food; sub-factor 2: the anticipation of positive reinforcement that may result from eating; sub-factor 3: the anticipation of relief from negative states and feelings as a result of eating; sub-factor 4: lack of control over eating; sub-factor 5: thoughts or preoccupation with food. Sub-factor 6 pertains to craving as a physiological state; sub-factor 7 addresses emotions that may be experienced before or during food cravings or eating; sub-factor 8 involves cues that may trigger food cravings; and sub-factor 9 relates to guilt from cravings and/or for giving in to them. All items in the scale have a 6-point Likert type, with responses categorized as follows: 6 = Always, 5 = Mostly, 4 = Frequently, 3 = Sometimes, 2 = Rarely, 1 = Never. An increase in the individual’s score on the scale indicates the development of excessive food cravings. The Cronbach’s alpha internal consistency coefficient was 0.97 (38).

#### 2.2.5 Emotional Eater Questionnaire (EEQ)

Garaulet et al. developed a ten-item questionnaire to evaluate the influence of emotions on eating behavior (39). The EEQ comprises three sub-dimensions: disinhibition (inability to control eating), food type (types of food), and guilt (guilt). Responses are provided on a Likert-type scale with four options: ‘0’ Never, ‘1’ Sometimes, ‘2’ Usually, and ‘3’ Always. The scale has no reverse items. On the scale, “0” represents the lowest total score and “30” represents the highest possible score. Higher scores on the questionnaire indicate an elevated level of emotional eating behavior. Arslantaş et al. examined the validity and reliability of the Turkish translation of the EEQ (40). The Cronbach’s alpha coefficient for the EEQ was 0.84, whereas the coefficients for “disinhibition,” “type of food,” and “guilt” were 0.81, 0.57, and 0.64, respectively (39).

### 2.3 Statistical analysis

The data were computerized and analyzed utilizing the IBM Statistical Package for the Social Sciences (SPSS) Statistics 22 (Armonk, NY, USA). The characteristics of participants, both general and related to the menstrual cycle, were assessed through descriptive-depictive analyses, including number, percentage, mean, and standard deviation. The normal distribution of the data was evaluated using visual methods (histogram and probability plots), the skewness and kurtosis, and statistical methods (Shapiro–Wilk test). The descriptive statistics were displayed as mean and standard deviation for numerical variables and frequency and percentage for categorical variables. An independent t-test was employed for the normally distributed data. The Mann–Whitney U test and the Kruskal-Wallis test were employed to analyze non-normally distributed data. Chi-square tests were employed for categorical data analysis. The Spearman correlation coefficient was utilized to represent the relationships between numerical variables. Multiple linear regression using an enter method was used to study the factors associated with hedonic hunger and craving for food tendencies. The statistical significance level was set at *p* < 0.05.

## 3 Results

Table 1 presents students’ general and menstrual cycle characteristics as shown by PMSS scores. The mean age of students without PMS was 21.03 ± 4.60 years, whereas for those with PMS, it was 21.10 ± 1.57 years (*p* < 0.05). Most students are in the second and third grades (29.0 and 28.0%, respectively). A significant number of students (83.6%) live in dormitories. Approximately two-thirds of students’ income equals their expenses (*p* < 0.05). The mean age of menarche among students was 13.08 ± 1.17 years, and they had a regular menstrual cycle with periods ranging from 22 to 34 days [PMS (−), 84.20%; PMS (+), 79.30%]. The duration of menstruation ranged from 3 to 7 days [PMS (−), 92.10%; PMS+, 92.30%]. Students with PMS reported a greater incidence of menstrual pain during their menstrual cycle (*p* < 0.05). No significant differences were observed among students regarding the number of main meals and snacks, smoking status, and alcohol consumption (*p* > 0.05). The mean BMI of the students was 23.3 ± 4.07 [PMS (−), 24.15 ± 4.81; PMS (+), 23.06 ± 3.87, respectively] (*p* > 0.05). There was no statistically significant difference between the students with PMS (−) and PMS (+) in terms of fat mass, total body water, and fat-free mass (*p* > 0.05).

**Table 1:** General and menstrual cycle characteristics of students based on PMSS scores.

Characteristics	PMS (−) (*n* = 38)	PMS (+) (*n* = 169)	Overall (*n* = 207)	*p*
Age (years)	21.03 ± 4.60	21.10 ± 1.57	21.09 ± 2.41	**0.016**
Grade of education				
1st	15 (39.50)	34 (20.1)	49 (23.70)	0.053
2nd	11 (28.90)	49 (29.0)	60 (29.00)	
3rd	6 (15.80)	52 (30.8)	58 (28.00)	
4th	6 (15.80)	34 (20.1)	40 (19.30)	
Living situation				
Living at dormitory	30 (78.90)	143 (84.60)	173 (83.60)	0.690
Living with flatmates at home	2 (5.30)	6 (3.60)	8 (3.90)	
Living with family at home	6 (15.80)	20 (11.80)	26 (12.60)	
Monthly income				
Income < expenses	2 (5.30)	31 (18.30)	33 (15.90)	**0.023**
Income = expenses	27 (71.10)	128 (75.70)	155 (74.90)	
Expenses > income	9 (23.70)	10 (11.80)	19 (9.20)	
Having chronic diseases				
Yes	8 (21.10)	25 (14.80)	33 (15.90)	0.341
No	30 (78.90)	144 (85.20)	174 (84.1)	
Menarche age (years)	13.00 ± 0.99	13.10 ± 1.21	13.08 + 1.17	0.530
Menstrual cycle (day)				
< 21	1 (2.60)	20 (11.8)	21 (10.10)	0.195
22–34	32 (84.20)	134 (79.3)	166 (80.20)	
> 35	5 (13.2)	15 (8)	20 (9.70)	
Duration of menstruation (day)				
3–7	35 (92.10)	156 (92.3)	191 (92.30)	0.966
> 7	3 (7.90)	13 (7.7)	16 (7.70)	
Menstrual pain				
Yes	17 (44.70)	138 (81.7)	155 (74.90)	**<0.001**
No	21 (55.30)	31 (18.3)	52 (25.10)	
Smoking status				
Never smoker	35 (92.10)	139 (82.2)	174 (84.10)	0.134
Current smoker	3 (7.90)	30 (17.8)	33 (15.90)	
Alcohol consumption				
Yes	–	5 (3.00)	5 (2.40)	0.283
No	38 (100.00)	164 (97.00)	202 (97.60)	
Number of main meals	2.42 ± 0.50	2.43 ± 0.50	2.43 ± 0.52	0.890
Number of snacks	1.39 ± 0.75	1.65 ± 0.67	1.60 ± 0.70	0.104
BMI (kg/m^2^)	24.15 ± 4.81	23.06 ± 3.87	23.3 ± 4.07	0.300
BMI classification				
Underweight	2 (5.30)	14 (8.30)	16 (7.70)	0.337
Normal	22 (57.90)	108 (63.90)	130 (62.80)	
Overweight	9 (23.70)	38 (22.50)	47 (22.70)	
Obese	5 (13.20)	9 (5.30)	14 (6.80)	
Fat mass (%)	28.89 ± 6.87	28.83 ± 12.57	28.86 ± 11.72	0.506
Total body water (kg)	52.83 ± 4.79	53.43 ± 5.21	53.32 ± 5.13	0.522
Fat-free mass (kg)	41.83 ± 5.81	40.90 ± 4.17	41.07 ± 4.51	0.831

*p* < 0.05.

BMI, body mass index; PMS, premenstrual syndrome.

Bold values denote statistical significance at the *p* < 0.05 level.

**Figure 1:**
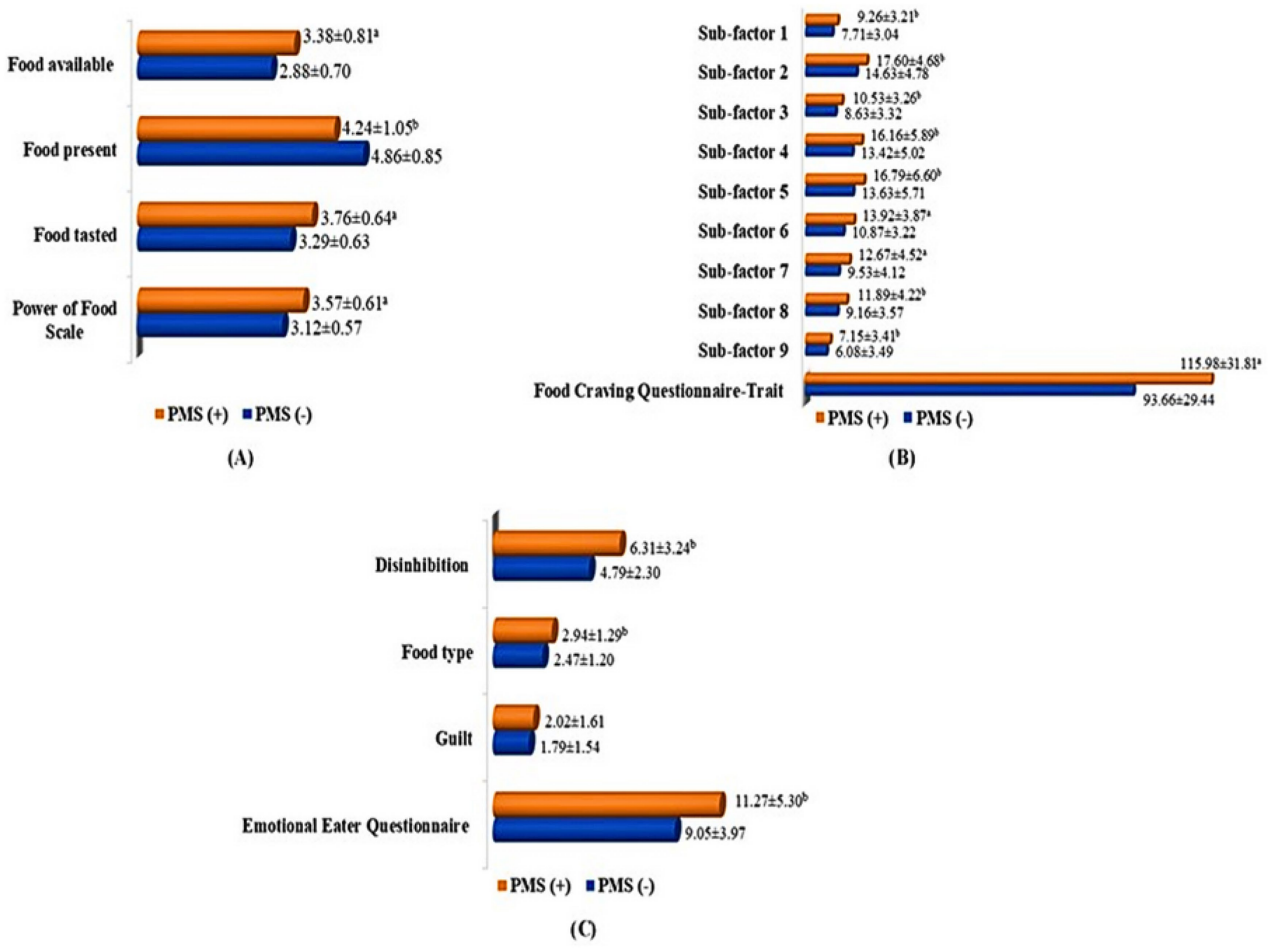
Power of Food Scale, Food Craving Questionnaire-Trait, and Emotional Eater Questionnaire total and subscale scores among students with PMS (−) and PMS (+). **(A)** Power of Food Scale, food available, food present, and food tasted scores in students with PMS (−) and PMS (+); **(B)** Food Craving Questionnaire-Trait and sub-factor 1, 2, 3, 4, 5, 6, 7, 8, 9 scores students with PMS (−) and PMS (+); **(C)** Emotional Eater Questionnaire, disinhibition, food type, and guilt scores in students with PMS (−) and PMS (+). a. *p* < 0.001; b. *p* < 0.05.

Figure 1 details the PFS-Tr, FCQ-T, and EEQ scores among students with PMS (−) and PMS (+). The PFS-Tr and subscales were significantly higher in the PMS (+) group (*p* < 0.001), except for the food presence subscale. In students with PMS (+), the FCQ-T and sub-factor scores were significantly higher (*p* < 0.001; *p* < 0.05). The emotional eating behaviors of students analysis revealed statistically significant increases in disinhibition, food type, and EEQ score among students with PMS (+). Compared to PMS (−) students, PMS (+) students had statistically higher EEQ, food type, and disinhibition scores (*p* < 0.05).

There was a positive correlation between the PFS-Tr total score and the total score of the PMSS, sub-factor scores of anxiety, fatigue, irritability, depressive thoughts, pain, appetite changes, and bloating. A positive correlation was observed between the total score of the FCQ-T and the total score of the PMSS, as well as the sub-factor scores for anxiety, fatigue, irritability, depressive thoughts, pain, appetite changes, sleep changes, and bloating. A positive correlation was found between the total score of the EEQ and the total score of the PMSS, the sub-factor scores for anxiety, fatigue, irritability, and bloating (*p* < 0.05; *p* < 0.001; Table 2).

**Table 2:** Evaluation of the relationship between PMSS, PFS-Tr, FCQ-T, and EEQ.

	PFS-Tr total score	FCQ-T total score	EEQ total score
	r	r	r
PMSS total score	0.293^**^	0.330^**^	0.244^**^
Depressive mood	0.096	0.124	0.114
Anxiety	0.154^*^	0.154^*^	0.164^*^
Fatigue	0.233^**^	0.247^**^	0.149^*^
Irritability	0.283^**^	0.277^**^	0.165^*^
Depressive thoughts	0.181^*^	0.234^**^	0.130
Pain	0.187^*^	0.220*	0.121
Appetite changes	0.555^**^	0.390^**^	0.346^**^
Sleep changes	0.101	0.205^*^	0.105
Bloating	0.184^*^	0.250^**^	0.204^*^

Spearman or Pearson correlation coefficient (r), ^*^p < 0.05, ^**^p < 0.001.

PMSS, premenstrual syndrome scale; PFS-Tr, Power of food scale-Turkish version; FCQ-T, Food Craving Questionnaire-Trait; EEQ, Emotional Eater Questionnaire.

When the factors (BMI, PMSS, FCQ-T, and EEQ total scores) related to PFS-Tr total scores were evaluated with multiple linear regression analysis, the model was significant (*R*^2^: 0.426, *p* < 0.001). FCQ-T was related to the PFS-Tr total score (*p* < 0.001). Higher cravings for food predicted increased hedonic hunger tendencies (Table 3). When the factors (BMI, PMSS, PFS-Tr, and EEQ total scores) related to FCQ-T total scores were evaluated with multiple linear regression analysis, the model was significant (*R*^2^: 0.614, *p* < 0.001). FCQ-T total score was related to PMSS, PFS-Tr, and EEE-Q total scores; however, BMI was unrelated (*p* > 0.05) (Table 4). A higher PMSS, increased craving for food, and emotional eating predicted increased craving (*p* < 0.05).

**Table 3:** Multiple linear regression analysis for Power of Food Scale-Tr prediction.

Model	*B*	PFS-Tr total score					
		SE	*Β*	*t*	*p*	Lower bound	Upper bound
Constant	2.183	0.269		8.111	0.000	1.652	2.713
BMI	−0.011	0.009	−0.069	−1.153	0.250	−0.028	0.007
PMSS total score	0.001	0.001	0.049	0.852	0.395	−0.002	0.004
FCQ-T total score	0.013	0.001	0.658	9.104	<0.001	0.010	0.015
EEQ total score	−0.001	0.009	−0.010	−0.140	0.888	−0.019	0.016
	*R*^2^:0.426 *p* < 0.001						

*p* < 0.05.

BMI, body mass index; EEQ, Emotional Eater Questionnaire; FCQ-T, Food Craving Questionnaire-Trait; PFS-Tr, Power of food scale-Turkish version; PMSS, premenstrual syndrome scale.

**Table 4:** Multiple linear regression analysis for Food Craving Questionnaire-Trait prediction.

Model	B	FCQ-T total score					
		SE	*β*	*t*	*p*	Lower bound	Upper bound
Constant	−31.907	13.079		−2.439	0.016	−57.696	−6.117
BMI	0.693	0.389	0.087	1.783	0.076	−0.073	1.459
PMSS total score	0.147	0.057	0.121	2.602	0.01	0.036	0.259
PFS-Tr total score	23.104	2.538	0.442	9.104	<0.001	18.1	28.108
EEQ total score	2.554	0.335	0.404	7.621	<0.001	1.893	3.215
	*R*^2^:0.614 *p* < 0.001						

*p* < 0.05.

BMI, body mass index; EEQ, Emotional Eater Questionnaire; FCQ-T, Food Craving Questionnaire-Trait; PFS-Tr, Power of food scale-Turkish version; PMSS, premenstrual syndrome scale.

## 4 Discussion

To the best of our knowledge, this is the first study to evaluate the relationship between hedonic hunger, food cravings, emotional eating, and PMS among Turkish nursing students. Our main findings were: (i) 81.6% of the female nursing students experienced PMS, (ii) students with PMS had significantly higher overall scores in PFS-Tr, FCQ-T, and EEQ compared to PMS (−) students, (iii) a positive and significant correlation was observed between PMSS and PFS-Tr scores, (iv) there was a positive and significant correlation between PMSS and FCQ-T scores, (v) the PMSS and EEQ scores were positively and significantly correlated.

The majority of students in this research experienced PMS (81.6% of 207 participants). A study by Gürkan and Bilgili (41) of university students in Türkiye found the prevalence of PMS was 34.2, 47.3% by Çelik and Uskun (42), 49.2% by Turan et al., (43). Further research indicated higher rates: 70.7% by Çağlar and Oskay (44), 72.4% by Yılmaz (45), 80.5% by Akın and Erbil (46), 83.7% by Arslan et al. (22). A recent systematic review and meta-analysis carried out in Türkiye revealed that PMS affected 52.2% of women of reproductive age, with 50.3% of those being university students (13). A study by Nandakumar et al. (47) of health professional undergraduate and postgraduate students in India found the prevalence was 76.4%, while Al-Shahrani et al. (48) reported 64.9% in Saudi Arabia, Rezende et al. found (11) 46.9% in Brazil. The prevalence of PMS in women of reproductive age was 47.8% globally (49). These outcomes result from various factors, such as living in a dormitory, academic challenges, sleep habits, stress levels, financial situations, and dietary and lifestyle choices. The variations in prevalence observed across studies can be attributed to factors such as cultural and regional differences, differing exclusion criteria, study design, and methods of sample selection.

Some research have examined the correlation between PMS symptoms and lifestyle factors (50, 51). Family history, age, oral contraceptive use, stress, smoking, eating habits, BMI, and physical activity may influence menstruation symptoms (52–54). Studies indicated a link between high BMI and menstruation problems, and disordered eating was found to be much more prevalent in the PMS (+) group compared to the PMS (−) group (16, 55). Our study indicated that there were no significant differences between PMS (+) and PMS (−) individuals for any general or menstrual cycle features of students, except for monthly income status and menstrual pain. The PMS (+) group had a significantly greater number of females reporting menstrual pain compared to the PMS (−) group. Variations in methodologies, sample sizes, hormonal variations during PMS, alterations in neurotransmitter systems, inflammatory responses, and the interplay of individual psychosocial variables are believed to have impacted the research outcomes.

During the luteal phase and the initial few days of menstruation, many women have food cravings, and changes in appetite and eating behaviors are found between healthy women and those with PMS (56–59). Previous studies have indicated that females experience increased appetite, especially for sweets, with an increase in the consumption of various food types throughout all menstrual phases. Notably, there was a significant rise in cravings for sweets and junk foods during the premenstrual period (60, 61), a positive association between PMSS scores and high-calorie diets, sweets, and salty and fried snacks (24), a significant association between high FCQ-T scores and PMS symptoms (62), and food cravings and PMS symptoms and changes in appetite were higher in the PMS group (63). As expected in this study, PFS-Tr and FCQ-T scores were shown to positively correlate with PMSS total and several subscale scores. Our findings indicate that students with PMS exhibited significantly higher total scores in the PFS, as well as increased scores in the food available and food tasted subscales. Furthermore, the total score and subscale scores of the FCQ-T were observed to be significantly higher in students with PMS compared to those without PMS. The results of our study coincide with previous research, indicating that increased food cravings and hedonic hunger are prevalent during PMS, with a more pronounced relationship observed between excessive food cravings and PMS compared to hedonic hunger.

Studies have shown that eating disorders are associated with PMS, with notable differences in appetite and dietary behaviors between healthy women and those having PMS (59, 64). PMS is significantly linked to impaired eating behaviors, particularly among female university students (65, 66). A study of adolescents revealed that disordered eating was significantly more prevalent in the PMS group compared to the non-PMS group, with higher scores for emotional and uncontrolled eating observed in the PMS group (67). In another study, the premenstrual dysphoric disorder group, severe form of PMS, showed higher mean scores for emotional eating compared to those without PMS (19). Consistent with these studies, it was determined that there is a positive correlation between EEQ scores and PMSS scores among nursing students. Students with PMS exhibited higher total EEQ scores, as well as elevated mean scores for disinhibition and food type-the sub-dimensions of emotional eating-compared to non-PMS students. The biopsychosocial characteristics of PMS require the use of several methods of treatment for symptom management (68). In this context, several psychological intervention programs have been established for reducing emotional eating behavior, with certain methods demonstrating efficacy. A research by Başoğul and colleagues shown that cognitive behavioral therapy significantly reduced both psychological and physical symptoms in women with PMS. Mindfulness-based therapies have been substantially correlated with PMS symptoms and may effectively decrease these symptoms (69, 70). Cognitive behavioral therapy and mindfulness-based therapies are considered helpful psychological interventions for mitigating emotional eating behaviors in women with PMS.

Hormone fluctuations during the menstrual cycle have a major impact on the hedonic aspect of eating behavior. Cyclical variations in estrogen and progesterone, essential hormones regulating the female reproductive system and menstrual cycle, are believed to significantly influence this process (71, 72). Estradiol, a steroid hormone classified as an estrogen, influences serotonin levels through modifications in the expression of 5-HT 2A receptors and serotonin transporter genes in brain areas linked to behavior and emotion (73). Women experiencing PMS exhibit serotonin deficits during the luteal phase of the menstrual cycle, coinciding with a decline in estradiol levels, which indicates a potential heightened sensitivity to the influence of estradiol on serotonin regulation (74). Changes in progesterone and allopregnanolone, the primary metabolite of progesterone, have also been associated with PMS. preliminary research suggest that decreasing progesterone levels in the late luteal phase correlate with elevated anxiety and changes in *γ*-aminobutyric acid (GABA) receptor function. In women experiencing PMS, it is suggested that a reduction in GABA (A) receptor sensitivity to neuroactive steroids, such as progesterone, and/or diminished levels of allopregnanolone could lead to symptoms of depression and anxiety (75, 76). Recent studies suggest a connection between brain-derived neurotrophic factor (BDNF) and the development of PMS, though its precise function remains to be clarified. While BDNF levels vary over the menstrual cycle, women with PMS have a declining trend in plasma BDNF throughout the ovarian cycle, contrasting with the rising trend seen in women without PMS (77–79).

The two primary phases of the menstrual cycle in women are the luteal and follicular. Increased homeostatic (related to energy demands) and hedonic (related to pleasure) hunger determinants may be implicated in the decrease of oestrogen levels during the luteal phase (80). Women exhibit distinct brain activity patterns in response to dietary cues across various periods of the menstrual cycle. It has been demonstrated that the orbitofrontal cortex is more activated during the luteal phase than during the late follicular phase. As we all know, palatable food is a natural reward. This has been identified as significantly influencing energy intake and food cravings during the luteal phase through the activation of reward system pathways (81–83).

The present study had several strengths. Pregnant or breastfeeding individuals, those outside the menstrual cycle such as in early menopause, those with irregular menstrual cycles, those with physical or mental health issues, any confirmed psychiatric disorders, or women prescribed psychiatric medication by a physician were excluded from the study. The accuracy of the data obtained, together with the low risk of bias, is a notable strength when considering the exclusion criteria. According to the existing literature, an initial study of the relationship between hedonic hunger, food cravings, emotional eating, and PMS was conducted among nursing students. This study has some limitations. First, the cross-sectional study design complicates the determination of cause-effect relationships between independent and dependent variables. Second, the participants consist only of nursing students from one university, hence constraining generalizability. Third, data collection included self-reporting, which introduces the potential for recall bias and inaccurate reporting. Finally, there is a lack of confounder information.

## 5 Conclusion and recommendations

Our study assessed the prevalence of PMS, menstrual cycle features, and anthropometric measures among university students, and elucidated the correlation between hedonic hunger, food cravings, emotional eating, and PMS. PMS was a problem experienced by most of the students (81.6%). Hedonic hunger, food cravings, emotional eating, and overall PMSS scores, along with many sub-scores, exhibited a significant correlation among nursing students. Food cravings were the most strong indicator of hedonic hunger, while hedonic hunger, PMS, and emotional eating all had important roles in predicting food cravings. According to the findings, nursing students who have PMS are more likely to have increased hedonic hunger, desires for food, and emotional eating tendencies. The intricate interplay among these factors indicates that strategies aimed at encouraging healthy eating behaviors should take into account the diverse aspects of these correlations. Further studies should concentrate on investigating more mediating factors that might affect the correlation between PMS and eating habits. More research on strategies for managing eating habits during PMS might be beneficial. Furthermore, replicating these findings across many populations may enhance their generalisability and facilitate the development of similar interventions for various demographic groups.

## Data Availability

The raw data supporting the conclusions of this article will be made available by the authors, without undue reservation.
